# Use of immunoinformatics and the simulation approach to identify *Helicobacter pylori* epitopes to design a multi-epitope subunit vaccine for B- and T-cells

**DOI:** 10.1186/s12896-023-00814-5

**Published:** 2023-09-27

**Authors:** Zahra Ahmadzadeh Chaleshtori, Ali Asghar Rastegari, Hashem Nayeri, Abbas Doosti

**Affiliations:** 1 Department of Biochemistry, Falavarjan Branch, Islamic Azad University, Isfahan, Iran; 2 Department of Molecular and Cell Biochemistry, Falavarjan Branch, Islamic Azad University, Isfahan, Iran; 3 Biotechnology Research Center, Shahrekord Branch, Islamic Azad University, Shahrekord, Iran

**Keywords:** *Helicobacter pylori*, Immunoinformatics, Vaccine, *LeoA*, *IceA1*, *IceA2*

## Abstract

**Background:**

*Helicobacter pylori* cause a variety of gastric malignancies, gastric ulcers, and cause erosive diseases. The extreme nature of the bacterium and the implantation of this bacterium protects it against designing a potent drug against it. Therefore, employing a precise and effective design for a more safe and stable antigenic vaccine against this pathogen can effectively control its associated infections. This study, aimed at improving the design of multiple subunit vaccines against *H. pylori*, adopts multiple immunoinformatics approaches in combination with other computational approaches.

**Results:**

In this regard, 10 HTL, and 11 CTL epitopes were employed based on appropriate adopted MHC binding scores and c-terminal cut-off scores of 4 main selected proteins (*APO*, *LeoA*, *IceA1*, and *IceA2*). An adjuvant was added to the N end of the vaccine to achieve higher stability. For validation, immunogenicity and sensitization of physicochemical analyses were performed. The vaccine could be antigenic with significantly strong interactions with TOLK-2, 4, 5, and 9 receptors. The designed vaccine was subjected to Gromacs simulation and immune response prediction modelling that confirmed expression and immune-stimulating response efficiency. Besides, the designed vaccine showed better interactions with TLK-9.

**Conclusions:**

Based on our analyses, although the suggested vaccine could induce a clear response against *H. pylori*, precise laboratory validation is required to confirm its immunogenicity and safety status.

## Background

As a helical-shaped bacterium, *Helicobacter pylori* (*H. pylori*) is a gram-negative bacterium mostly found on the inner surface of the stomach. *H. pylori* is the most common cause of multiple infections, such as gastric carcinoma, peptic ulcers, mucosa-associated lymphoid tissue (MALT), and peptic ulcers [1]. Based on genetic composition analysis*, *a notable heterogeneity was observed in the gene sequence and content of the *H. pylori* bacterium. Besides, as a versatile gene reservoir, it could be easily used as an eligible tool for bacterial adaptation when new conditions occur in different human hosts. The presence of *H. pylori* could be quickly approved through the continuous induction of inflammatory responses as well as tissue damage that can cause extra critical clinical disorders, such as peptic ulcers (PUs), chronic gastritis (CG), osteoporosis (OP), and gastric cancer [2, 3]. Given the dangers of *H. pylori*, controlling the infection with antibiotics may reduce the incidence of the disease. Nowadays, *Helicobacter pylori* infections can be treated using some antibiotics; however, there are some limiting factors to these treatments, such as high-cost het, patient reluctance to take the drug, and development of severe antibiotic resistance [4]. Therefore, there is a need for alternative treatment or prevention methods, such as vaccination. Attempts to develop a vaccine against *H. pylori* began in the early 1990s, with the knowledge that the infection caused by this bacterium was the leading cause of gastric ulcers. Despite research on the *H. pylori* vaccine with acceptable results in animal models and cell cultures, no vaccine has been licensed for human use [5, 6]. Based on recent reports, some of these factors may increase the risk of infections. Virulence factors of *H. pylori* could result in the production of pro-inflammatory cytokines dominant in gastric disorders and malignant proteins [1].

The *lnT* gene (Apolipoprotein N-acyltransferase) was first discovered in the gram-negative bacterium *Salmonella enterica*. However, the structure and *lnT* function were later studied in more detail in Escherichia coli. 1–3% of the bacterial genome contains genes that encode lipoproteins in the cell membrane. These lipoproteins can play vital roles, such as nutrient uptake, protection of cell wall integrity, protein secretion, cytoplasmic folding of proteins, and pathogenicity [7]. Lipoproteins are the significant components of the cell lining, being responsible for many essential functions of the cell. Powerful covalent bonds produce these components after translations in the context of "lipids," which are created by a sequential process consisting of three steps and controlled by three integral membrane enzymes. The final step of this process, creating the final mature lipoprotein, is the N-acylation of the terminal cysteine by Apolipoprotein N-acyltransferase (Lnt). This step is mainly unique to gram-negative bacteria. Synthesized proteins are pro-lipoproteins transported to the cytoplasmic membrane by the *Sec* (General secretion) or *Tat* (twin-arginine translocation) general secretory machinery [8].

The *lnT* gene encodes one of the significant coat lipoproteins of Helicobacter pylori, which stimulates immunogenicity in the host cell, producing antibodies. The cloning and antigenic properties of the *lnT* gene have been studied in other gram-negative bacteria, such as *E. coli* and *S. enterica* [9]. The *leoA* gene (labile enterotoxin output A) is one of the pathogens of *H. pylori* GTPase, which is encoded on pathogenicity islands and potentially increases toxin release through secretory vesicles. *LeoA* is known as a bacterial diamine-like protein. Diamine family proteins alter membranes and generally evolve by releasing heat-sensitive enterotoxin through the membrane vesicle, released from the bacterial cell's surface. There are few reports on the functional role of bacterial diamine proteins [10]. Membrane vesicles play a vital role in the protected function of the diamine family members. Reports show that this gene has the potential for immunization in the host body. Therefore, it has been introduced as a confirmed candidate for the assembly of a gene vaccine against *H. pylori* [11, 12].

After the isolation of *H. pylori* bacteria from peptic ulceration disease (PUD) and gastritis patients, the *iceA* gene was identified. *Helicobacter pylori* *iceA1* and *iceA2* genes are the two main alleles of *iceA* [13]. After bonding *H. pylori* to human epithelial cells, the expression of *iceA1* was upregulated. The iceA1 genotype caused both mucosal IL-8 expression and acute antral inflammation. The bonding of *iceA1* to gastric epithelial cells in vitro stimulates its transcription [14]. Many studies have proposed an association between the *iceA1* variant and PUD. However, no homology has been found between *iceA2* and other known genes. Although *iceA2* can cause asymptomatic gastritis and non-ulcer dyspepsia, its function has remained unknown [15]. More than 8% of patients had this gene in connection with gastritis, more than 8% had this gene in combination with mixed lesions, and more than 6% had the iceA1 gene in combination with peptic ulceration. However, the *iceA2* gene was less prevalent than the *iceA1* gene in patients with mixed lesions (10%), while gastritis and peptic ulceration were prevalent in 12.5%. None of the *iceA1* and *iceA2* genes was prevalent in patients with gastric carcinoma. Unexpectedly, in patients with gastric carcinoma, *iceA1*, and *iceA2* genes were more prevalent. The higher rate of the prevalence of these genes in these patients could be a sign warning against the higher risk of developing gastric carcinoma. There was a lower prevalence of positive cases of the two *iceA* alleles among patients with gastritis, ulcers, and mixed lesions [16]. Immunoinformatics is one of the fastest, most precise, and most reliable approaches to developing vaccines against virulent pathogens. Given the advantages and feasibility of designing vaccines using immunoinformatics approaches [1], our study aimed to design a multi-epitope subunit vaccine against *H. pylori*. The present study used a wide range of antigenic proteins from the *H. pylori* proteome to design B- and T-cell epitopes. For this purpose, MHC-II binding epitopes (HTL) were used as a predictive method, and a vaccine composed of many epitopes was produced in the end. Additionally, molecular docking for the drug design, molecular dynamics for stability profiling, ''in silico expression analysis'' for validating short DNA sequences, and agent-based computational modelling were employed to check the immune response reaction and stability stimulated by the final vaccine produced.

## Results

### Retrieval of *Helicobacter pylori* protein sequences

The amino acid sequences of *leoA*, I*ceA*1-2, and *APO* of *H. pylori* were retrieved. This bacterium was retrieved from the gene bank to develop a multi-epitope subunit vaccine to evoke immune reactions against the *H. pylori* infection. These proteins were selected based on their reliability and target efficacy.

### Antigenicity of selected *H*. *pylori* proteins

The antigenicity of the proteins was determined using the Vaxijen2.0 server. Accordingly, the results showed that all selected proteins in this study had antigenic attributes. This server calculated scores 0.4356, 0.3076, 0.5319, and 0.4370 for *APO*, *leoA*, *IceA1*, and *IceA2*, respectively [17].

### Prediction of cytotoxic and HTL epitopes

Based on NetCTL 1.2, 37 CTL epitopes were anticipated for the selected proteins, of which only 13 epitopes with a non-allergenic nature and a binding score were considered for this study. Similarly, using the IEDB MHC-II server resulted in CTL epitopes for the selected proteins, as Table 1 (bold columns) shows.

**Table 1:** Cytotoxic T-lymphocyte epitopes identified

Protein (GenBank ID)	Residue no	Peptide sequence	MHC binding affinity	Rescale binding affinity	C-terminal cleavage affinity	Transport affinity	Prediction score	MHC-I binding	ML score	AlgPred2 prediction
***APO*** **(ACX97396.1)**	1	HSANFSTSY	0.7246	3.0765	0.9736	2.939	3.3694	Yes	0.31	Allergen
	22	YVNAVLDAY	0.3384	1.437	0.735	2.996	1.697	Yes	0.32	Allergen
	34	FSDFTLDDF	0.3589	1.5237	0.2645	2.281	1.6774	Yes	0.34	Allergen
	82	YSDFTYLLP	0.371	1.5751	0.0253	0.081	1.583	Yes	0.38	Allergen
	62	LSAQNLKKY	0.3036	1.2892	0.4645	2.881	1.503	Yes	0.32	Allergen
	**97**	**ALVYGVLFY**	**0.2362**	**1.003**	**0.9535**	**3.175**	**1.3048**	Yes	**0.28**	**Non-allergen**
	1	GVLFYLLLY	0.1966	0.8348	0.9298	2.85	1.1168	Yes	0.3	Non-allergen
	42	FTFRPLICY	0.1862	0.7908	0.9743	2.781	1.076	Yes	0.32	Allergen
	82	TLQRTLLKY	0.181	0.7683	0.967	2.746	1.0507	Yes	0.31	Allergen
	34	LVPDSFFSY	0.1726	0.733	0.9773	2.996	1.0294	Yes	0.34	Allergen
	**92**	**IVVLIALVY**	**0.1673**	**0.7103**	**0.6759**	**3.212**	**0.9723**	**Yes**	**0.27**	**Non-allergen**
	18	VSSVYVNAV	0.1889	0.8022	0.351	0.308	0.8702	Yes	0.32	Allergen
	59	NSAFALGFF	0.1663	0.706	0.0585	2.761	0.8528	Yes	0.35	Allergen
*LeoA* **(ACX97803.1)**	113	FSDLICQLL	0.2635	1.1187	0.9296	0.672	1.2917	Yes	0.34	Allergen
	**104**	**NLKEFITRY**	**0.1663**	**0.706**	**0.9786**	**2.988**	**1.0022**	**Yes**	**0.22**	**Non-allergen**
*IceA1* **(AAL59391.1)**	128	VSDSNTQTF	0.466	1.9786	0.8282	2.593	2.2325	Yes	0.32	Allergen
	166	ATKIPGNYY	0.2931	1.2443	0.9723	2.906	1.5355	Yes	0.46	Allergen
	25	SAIEFVGKY	0.176	0.7472	0.9769	3.146	1.051	Yes	0.47	Allergen
	165	DATKIPGNY	0.1545	0.6561	0.9429	2.52	0.9235	Yes	0.42	Allergen
	108	NSENAQIEI	0.1694	0.7191	0.7521	0.438	0.8539	Yes	0.32	Allergen
	182	EYDGCVGCY	0.1264	0.5366	0.9674	2.963	0.8298	Yes	0.42	Allergen
	214	YGEGYQIGY	0.1361	0.578	0.8502	2.464	0.8288	Yes	0.43	Allergen
*IceA2* **(AAG49536.1)**	84	YSFVLNQNY	0.5255	2.2314	0.8765	3.005	2.5131		0.31	Allergen
	207	LSLNANEVY	0.4015	1.7049	0.9707	2.848	1.9929		0.33	Allergen
	**24**	**SMNYIGSKY**	**0.3506**	**1.4885**	**0.9292**	**3.137**	**1.7848**		**0.24**	**Non-allergen**
	**175**	**KVANTASVY**	**0.2678**	**1.1369**	**0.9308**	**3.048**	**1.4289**		**0.24**	**Non-allergen**
	**178**	**NTASVYGAF**	**0.2666**	**1.1321**	**0.3672**	**2.721**	**1.3232**		**0.24**	**Non-allergen**
	**76**	**VISNDLEYY**	**0.2121**	**0.9005**	**0.8903**	**3.212**	**1.1947**		**0.24**	**Non-allergen**
	154	SQNIDNHSY	0.2084	0.8847	0.9431	3.007	1.1766		0.37	Allergen
	156	NIDNHSYYF	0.2047	0.8693	0.9299	2.475	1.1325		0.37	Allergen
	75	KVISNDLEY	0.1832	0.7779	0.8021	3.38	1.0672		0.37	Allergen
	308	ETEIKNILK	0.2192	0.9308	0.4751	0.149	1.0095		0.37	Allergen
	247	HLLNTIAAY	0.1594	0.6768	0.9698	2.819	0.9632		0.36	Allergen
	**133**	**FSETNAQKI**	**0.1936**	**0.8221**	**0.1326**	**0.284**	**0.8562**		**0.25**	**Non-allergen**
	**112**	**VALKKGFIY**	**0.1303**	**0.5531**	**0.9487**	**3.111**	**0.851**		**0.28**	**Non-allergen**
	**313**	**NILKKYGAY**	**0.1312**	**0.557**	**0.8046**	**3.209**	**0.8381**		**0.3**	**Non-allergen**
	**124**	**SLGGSSRQY**	**0.1113**	**0.4727**	**0.9647**	**2.782**	**0.7565**		**0.24**	**Non-allergen**

HTL epitopes predicted by the server included the seven sets of HLAs, including HLA-DRB1 * 03:01, HLA-DRB1 * 07:01, HLA-DRB1 * 15:01, HLA-DRB3 * 01:01, HLA-DRB3 * 02:02, HLA-DRB4 * 01:01, and HLA-DRB5 * 01:01. Table 2 shows these HTL epitopes. The server predicted different numbers of HTL epitopes, such as one for *leoA* and three for *IceA1*, *IceA2*, and *APO*, each having been at different positions.

**Table 2:** Helper T-Lymphocyte epitopes and their scores anticipated using the IEDB MHC class II server

Protein	Allele	Start	End	Length	Method	Peptide	Percentile rank
***APO***	HLA-DRB4*01:01	346	360	15	Consensus (comb.lib./smm/nn)	PLICYEGTSKAAYSN	64
	HLA-DRB1*03:01	367	381	15	Consensus (smm/nn/sturniolo)	IVMSNNAWFSPSIEP	19
	HLA-DRB1*03:01	267	281	15	Consensus (smm/nn/sturniolo)	ILIGTLRTQGYNLYN	35
***LeoA***	HLA-DRB1*07:01	34	48	15	Consensus (comb.lib./smm/nn)	QKKIKENGGKLTIIE	52
***IceA1* (AAL59391.1)**	HLA-DRB1*01:01	33	47	15	Consensus (comb.lib./smm/nn)	YQGLQLGNGGSWRNN	53
	HLA-DRB1*07:01	55	69	15	Consensus (comb.lib./smm/nn)	EFDKGQTPGNSIDRI	48
	HLA-DRB1*04:01	121	135	15	Consensus (smm/nn/sturniolo)	DLRVSDSNTQTFDDF	14
***IceA2* (AAG49536.1)**	HLA-DRB5*01:01	253	267	15	Consensus (smm/nn/sturniolo)	AAYTPFTPKGKTGLP	4.3
	HLA-DRB5*01:01	60	74	15	Consensus (smm/nn/sturniolo)	AGTGIVGRAFKKAVN	21
	HLA-DRB4*01:01	144	158	15	Consensus (comb.lib./smm/nn)	MRLKIEELKLSQNID	7.2

^*^It shows the difference of alleles

### Vaccine production

To design a unique vaccine meeting all existing standards regarding nontoxicity, antigenicity, non-allergenicity, and binding affinity, 4 HTL epitopes, four lymph cell epitopes, and 10 CTL epitopes were nominated. Also, human β-defensins were introduced to an adjuvant with GI: 159162606. Human beta-defensins are a family of epithelial cell derived antimicrobial peptides (AMPs) that protect mucosal membranes from microbial challenges. Therefore, it can be used as the best adjuvant to enhance the vaccine's performance. The defensive mechanisms of β-defensins include binding to negatively charged microbial membranes that cause cell death and chemoattraction of immune cells.

**Fig. 1:**
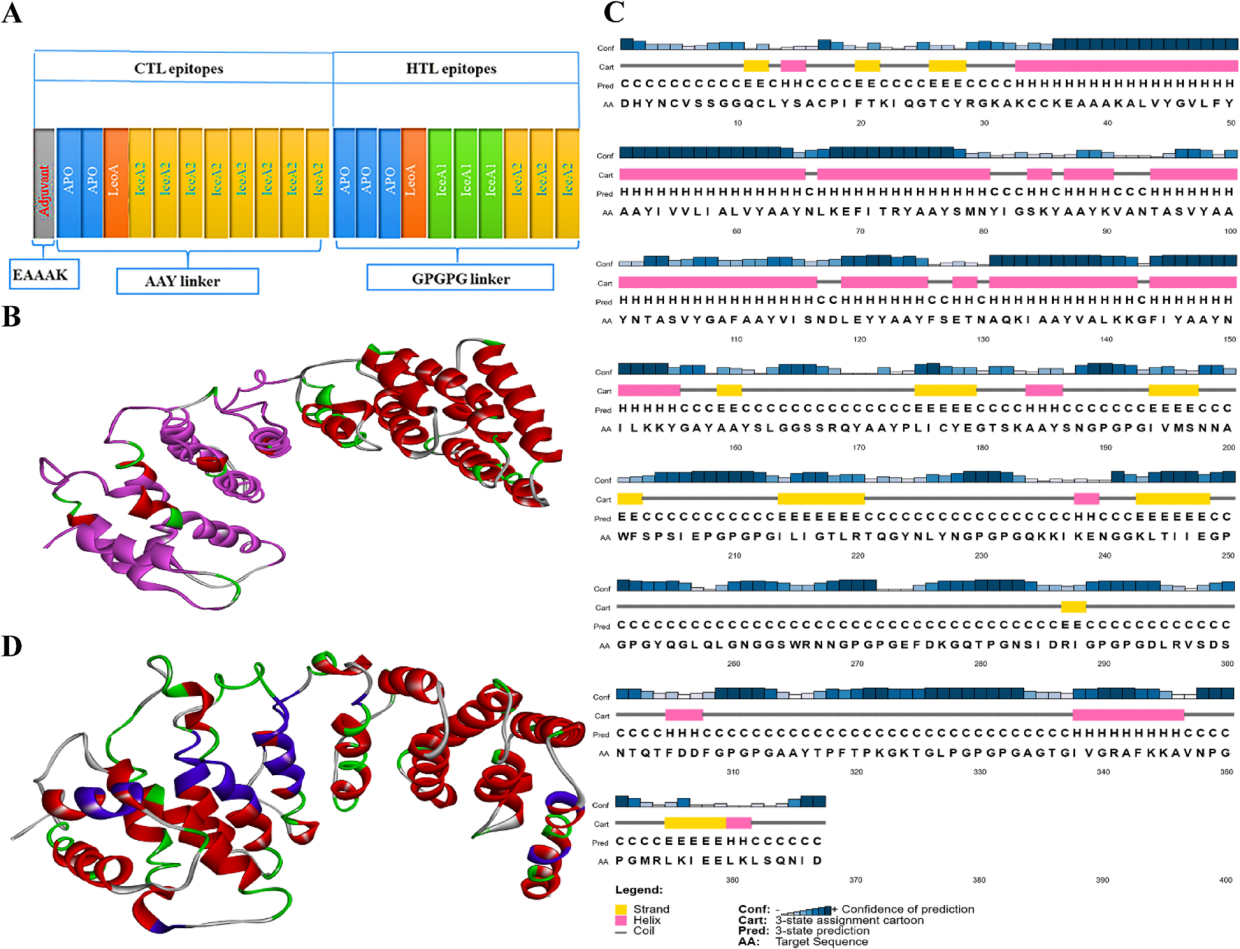
**A** Arrangement of the HTL and CTL epitopes in the final vaccine construct using different linkers. **B** B-cell epitopes as predicted by the BCPred server; in the 3D structure of the vaccine, linear B-cell epitopes are presented in magenta, while conformational B-cell epitopes are presented in pink. **C** Predicting the secondary structure and solubility of the vaccine construct; the results of the PSIPRED server for predicting the secondary structure of the vaccine construct showed 46.86% coils, 38.14% alpha-helices, and 10.35% beta-strands. **D** Multi-template modeling of the final 3D structure of the vaccine; the relevant elements are depicted in different colors, loops in sky blue, beta-sheets in colored blue, and helices in red

The ultimate multi-epitope vaccine was produced from 10 CTL and 13 HTL epitopes (shown in bold letters in Table 1) by joining a couple of AAY and GPGPG linkers. Besides, the adjuvant was attached to N-terminals to avoid vaccine degradation. Additionally, GPGPG and AAY linkers joined the HTL and CTL epitopes, while the EAAAK linker tended to bond with the adjuvant of the CTL epitopes. Figure 1A shows the ultimate vaccine structure.

### Prediction of B cell epitopes

To predict B-cell epitopes by the BCPred server, default parameters were applied. Accordingly, the server predicted 14 linear B-cell epitopes with a length of 20 amino acids and a score more excellent than 0.90. Figure 1B shows the predicted linear and conformational epitopes.

### Calculation of physiochemical properties

Some essential features were quantified, including allergenicity, instability, half-life, and relative molecular mass. The server calculated a score of -0.42 for the allergenicity of the last vaccine construct, thereby identifying the vaccine as non-allergenic. The default threshold was set as -0.4. The isoelectric point was determined to confirm the vaccine's fundamental nature. In addition, the server calculated a PI score of 9.23 and a mass of 38.718 kDa (MW). On the other hand, the in vitro half-life of the yeast E. coli (in mammals' reticulocytes) was calculated at > 30, > 20, and > 10 h. In addition, an instability index of 23.60 was obtained, confirming that the vaccine could remain stable under experimental conditions. Moreover, the aliphatic coefficient and GRAVY were calculated at 1.593 and -0.205, respectively.

### Prediction of secondary structure elements

The PSIPRED web server predicted the secondary structure of the vaccine protein. Additionally, the secondary structure of the elements was predicted at 46.86% coils, 38.14% α-helix, and 10.35% β-sheet (Fig. 1C).

### Modeling and refining the 3D structure

The I-TASSER web tool was utilized to model the final 3D structure of the multi-epitope vaccine. Figure 1D shows that the best fusion protein structure was obtained with a TM score of 0.56 ± 0.15, a C-score of -1.25, and an RMSD of 9.5 ± 4.6 Å.

### 3D structure validation

**Fig. 2:**
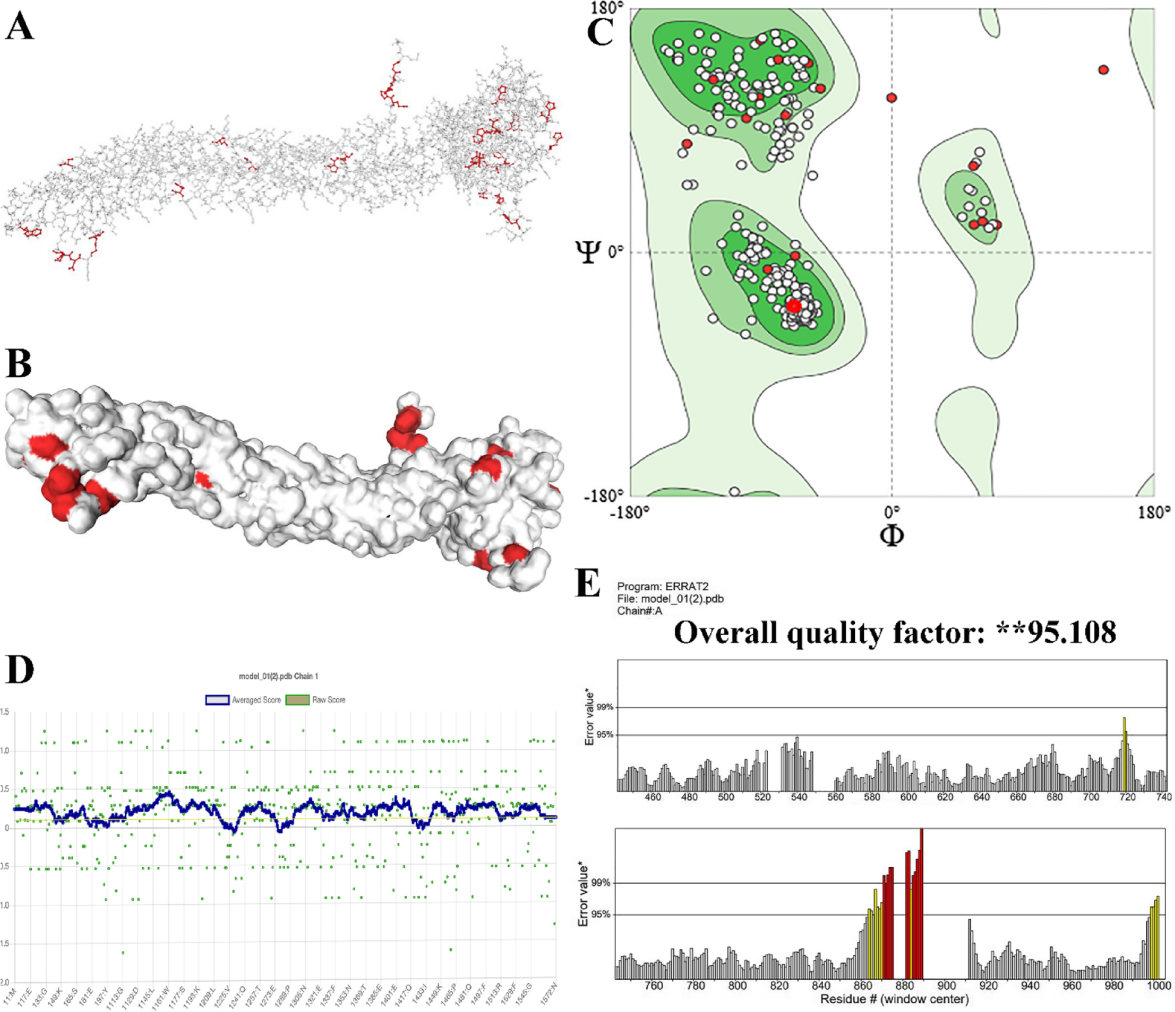
Analysis of the PROSA-web server and the Ramachandran plot; **A** and **B** shows 27 bad angels (A897 ILE-A898 ASN), A876 PHE, A911 TRP, (A725 PRO-A726 PRO), A566 ASP, A544 ASP, (A724 ILE-A725 PRO), (A900 GLY-A901 LEU), A896 SER, A549 PHE, A751 ASP, A898 ASN, (A583 ASN-A584 PRO), A833 ASP, A782 SER, A686 HIS, A556 HIS, A514 ASP, (A1003 GLU-A1004 PRO), A563 HIS, A512 TRP, (A536 ILE-A537 ASP), A595 ASP, (A1006 ARG-A1007 GLY), A524 HIS, A673 HIS); **C** shows amino acid distribution in desired (82.5% of residues), permissible (15% of residues), and impermissible (2.4% of residues) areas using the Ramachandran plot. **D**, **E** Validation tools like Verify 3D, and ERRAT was showed the quality of the tertiary structure of vaccine was **95.108

The refined 3D structure of the modeled multi-epitope vaccine was validated using RAMPAGE. The results showed that 82.5% of the vaccine residues were within the desired area, 15.0% within the permissible area, and only 2.4% within outlier areas. The quality of the model was further assessed using the ProSA web server, resulting in a Z-score of -0.67. Moreover, the ERRAT server calculated the confidence of 92.758% for the model. Figure 2 shows the plots obtained from RAMPAGE and PROSA web servers.

### Vaccine interaction analysis using TLR receptors

**Fig. 3:**
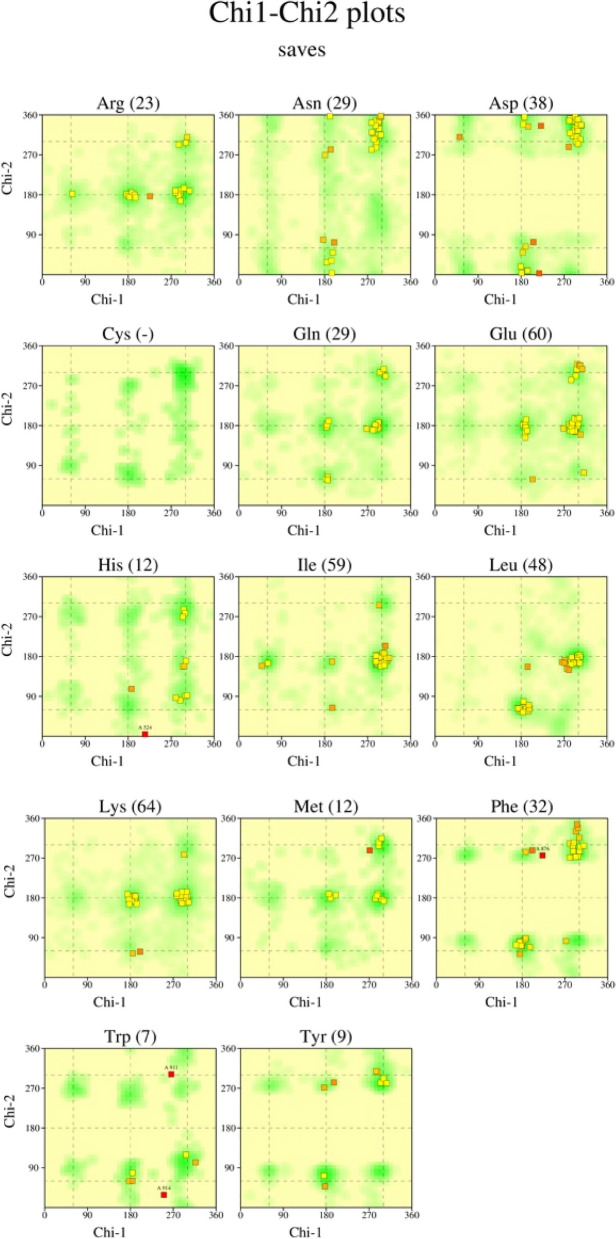
Chi-Chi 2 plots analysis. Numbers of residues were shown in brackets. Those in unfavorable conformation (score < -3.00) were labelled. Shading showed favorable conformation as obtain from an analysis of 163 structure at resulation 2.0A˚ or better

To improve prediction accuracy of the PatchDock server, several online tools were employed for the protein–protein docking of the vaccine. As Table 3 shows, the receptors used in this study included several TLR receptors (TLR-2, 4, 5, and 9). Based on the electrostatic complementarity and geometry of the protein’s surface, the Patchdock server ranked in the top 10 interaction models. At the same time, the global energy of the docked complexes was predicted by the FireDock server. In addition, the FireDock web tool was employed to refine and rescore top complexes. The results showed that binding to the TLR-9 complex produced a good global binding energy score (-23.59 kcal/mol), while the lowest binding affinity was obtained through docking with TLR-5 (-7.80 kcal/mol). Also, Chi-Chi 2 plots analysis was done (https://saves.mbi.ucla.edu/results?job=1348329&p=procheck). Numbers of residues were shown in brackets. Those in unfavorable conformation (score < -3.00) were labelled. Shading showed favorable conformation as obtain from an analysis of 163 structure at resulation 2.0A or better (Fig. 3).

**Table 3:** Results from docking with different toll-like receptors (TLR-2, 4, 5, and 9)

Protein name/ID	Global Energy	Attractive van der waals energy	Repulsive van der waals energy	Atomic contact energy (ACE) softened	Hydrogen bonded
**TLR-2 (6nig)**	-13.52	-23.48	9.10	5.49	-5.27
**TLR-4 (3fxi)**	-15.26	-29.25	16.65	-4.70	-1.56
**TLR-5 (3j0a)**	-7.80	-17.97	17.83	-0.65	-2.44
**TLR-9 (modeling)**	-23.59	-38.69	65.85	-13.88	-2.79

### MD simulation of TLR-vaccine complexes

**Fig. 4:**
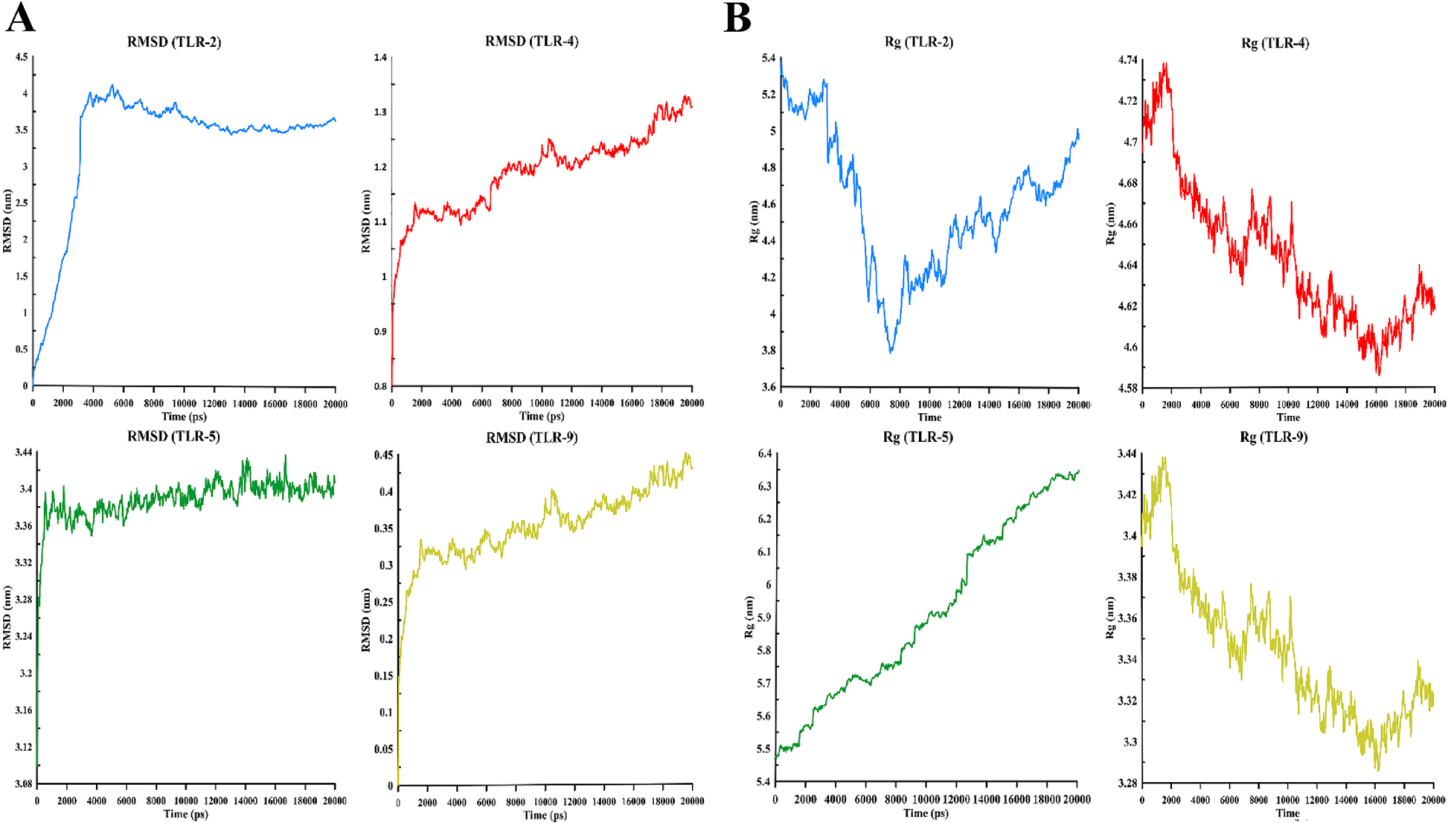
**A** Fluctuations and residual stability (i.e. RMSDs) of the whole system obtained from 20 ns of simulations. **B** The radius of gyration (Rg) of the whole systems obtained from 20 ns of simulations

MD simulations of the final docking vaccine complexes were performed to calculate fluctuations and residual stability using the selected receptors (TLR-2, 4, 5, and 9). Table 4 shows the results of these simulations. In addition, RMSF was assessed to quantify residual fluctuations; on the other side, RMSD was assessed to quantify the stability of each system (Fig. 4A).

**Table 4:** The average and standard deviations of the radius of gyration (Rg), area per residue, root-mean-square deviation (RMSD), root mean square fluctuations (RMSF) (Chain A includes; TLR-2, 4, 5 and 9, and Chain B includes the vaccine), the hydrogen bond between protein–protein, H-bond between protein-solvation (number), between the vaccine with the selected receptors (TLR-2, 4, 5 and 9)

Name	Distance (nm)	RMSD (nm)	Rg (nm)	RMSF (nm) Chain A	RMSF (nm) Chain B	Area per residue (ASA)(nm)	H-bond between protein–protein (number)	H-bond between protein-solvation (number)	Kinetic (kJ/mol)	Potential (kJ/mol)	PV (kJ/mol)	Temperature (K)
**TLR-9**	3.65 ± 0.06	0.44 ± 0.05	3.30 ± 0.02	0.14 ± 0.06	0.184 ± 0.07	376.85 ± 2.56	10.91 ± 2.76	1668.33 ± 57.94	363,101.6 ± 1095.39	-1,914,201 ± 1700.799	88.90 ± 0.12	300.00 ± 0.90
**TLR-4**	2.68 ± 0.06	1.35 ± 0.09	4.64 ± 0.02	0.48 ± 0.07	038 ± 0.07	469.86 ± 17.72	8.89 ± 2.77	1690 ± 66 ± 57.47	363,201.6 ± 1095.39	-1,915,966 ± 1699.99	140.45 ± 5.08	299.51 ± 0.90
**TLR-2**	2.25 ± 0.06	3.31 ± 0.90	4.59 ± 0.36	1.50 ± 0.30	1.35 ± 0.10	450.59 ± 17.13	46.42 ± 12.50	1141.22 ± 27.44	364,592.6 ± 941.47	-1,985,968 ± 60,391.54	1001.90 ± 8.43	300.02 ± 0.77
**TLR-5**	2.27 ± 0.16	3.38 ± 0.03	5.73 ± 0.14	1.61 ± 0.12	1.48 ± 0.07	476.12 ± 3.05	98.57 ± 10.86	1121.96 ± 25.03	364,359.8 ± 1099.48	-1,977,031 ± 1674.92	89.51 ± 0.12	299.91 ± 0.90

In total, 30 ns simulations were determined for all systems, reporting RMSD variables of 0.44 ± 0.05 nm for TLR-9, 1.35 ± 0.09 nm for TLR-4, 3.31 ± 090 nm for TLR-2, and 3.38 ± 0.03 nm for TLR-5. RMSD results showed that the complex TLR-9 with the vaccine was more stable over the simulation time and could establish better connections than other TLRs. The residual fluctuation (RMSF) results in Table 4 show that RMSF changes in TLR-2, 4, 5, and 9 were more significant than the vaccine structure, except for the TLK-9 vaccine complex. In addition, RMSF changes in the vaccine (0.184 nm) were more significant in the presence of Tlk-9 (0.14 nm), indicating that the structure of the vaccine in this complex was more flexible. Figure 4B shows the radius of the gyration (Rg) between the total vaccine complex and TLR-2, 4, 5, and 9. Rg shows the degree of compaction and the level of protein availability.

**Fig. 5:**
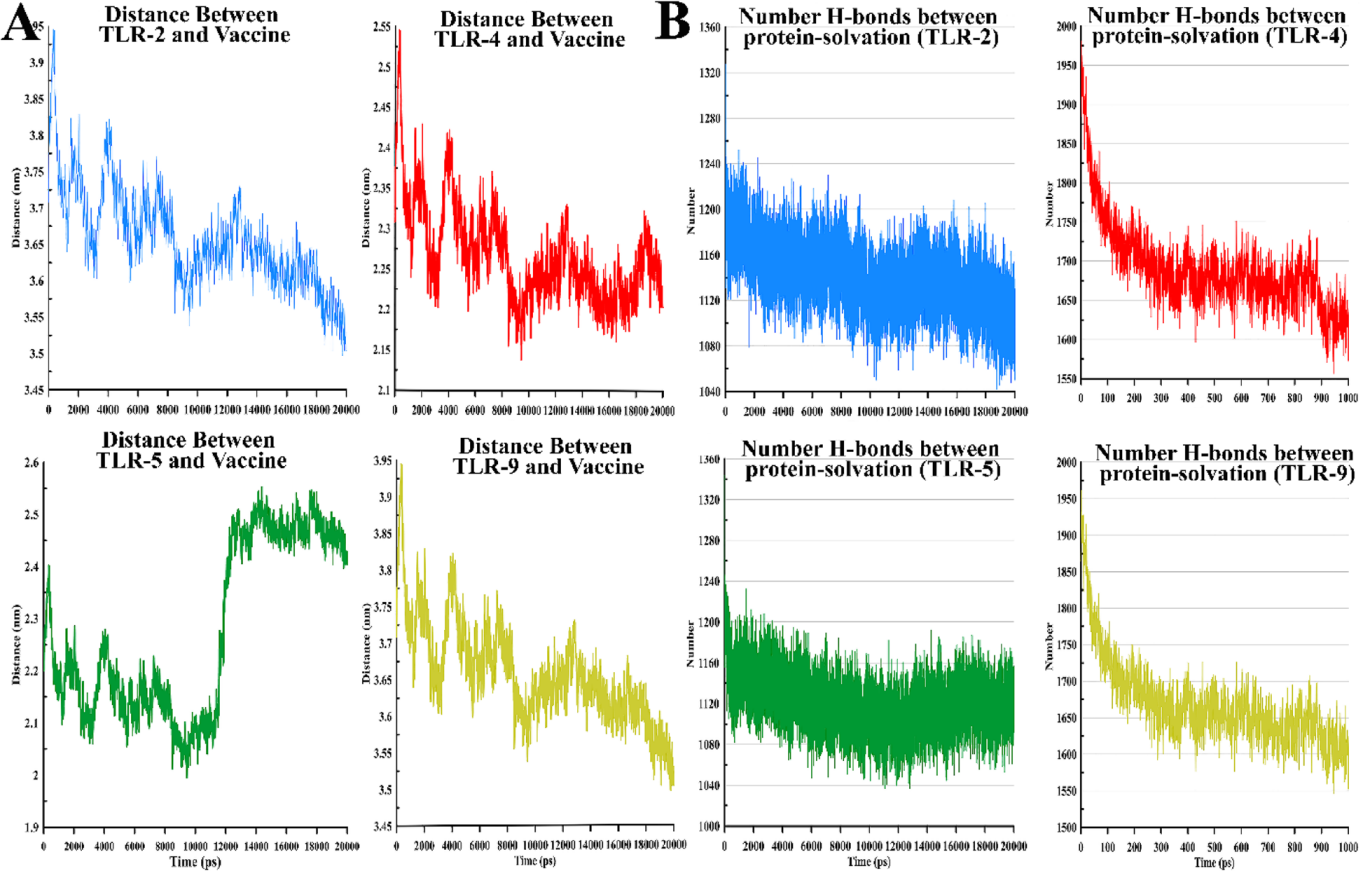
**A** Distance between the vaccine and proteins (TLR-2, TLR-4, TLR-5, and TLR-9) for the whole systems obtained from 20 ns of simulations. **B** Solvation and hydrogen bonds among proteins (TLR-2, TLR-4, TLR-5, and TLR-9) for the whole systems obtained from 20 ns of simulations

As the images show, TLK-4 and TLK-9 were more compact than TLK-2 and TLK-5 overtime during the simulation. In addition, the Rg value of TLK-9 (3.30 nm) was the smallest among TLK-2 (4.59 nm), TLK4 (4.64 nm), and TLK-5 (5.73 nm); accordingly, this indicates that the designed vaccine interacted better with TLK-9, being consistent with the docking results. Figure 5A shows the distance between TLR-2, 4, 5, and 9 and the vaccine at the nanometer scale. Fluctuations of the TLK-5 distance were more significant, while within a specific range, TLK-4 and TLK-9 decreased at the end of the simulation time. The distance between TLK-5 and the vaccine increased with the vaccine having little exposure to TLK-5.

As Fig. 5B shows, the number of hydrogen bonds between TLK-2 and TLK-5 was within the range of 1,160, while in TLK-4 and TLK-9 simulations for the first two nanoseconds, the number of hydrogen bonds started from 1,950 and then fluctuated within the range 1,650; this indicates that TLK-4 and TLK-9 had more solubility and could interact better.

## Discussion

Due to the dangers of *H. pylori*, controlling its infections with antibiotics may reduce the incidence of the disease. Some antibiotics are now available for treating *H. pylori* infections; however, high costs, patient unwillingness to regularly use the drug and the development of severe antibiotic resistance are among the factors limiting the adequacy of these treatments [4]. Hence, there is an urgent need for an alternative treatment or a prevention method, such as vaccination. Attempts to develop a vaccine against *H. pylori* began in the early 1990s, with the knowledge that the infection caused by this bacterium was the leading cause of gastric ulcers. Despite research on the *H. pylori* vaccine and acceptable results in animal models and cell cultures, no vaccine has been licensed for human use [5, 18]. In this regard, gene vaccines have attracted increasing attention due to their cost-effectiveness, effectiveness, and high immunogenicity in terms of humoral or cellular immunity, or even both [19, 20].

Contrary to classical approaches to vaccination, modern research has made it possible to produce "subunit vaccines" that accommodate microbial-specific protein sequences capable of effectively stimulating the reaction. Thanks to ample information on microbial genomes and proteomes, developing effective vaccines against various infections have been possible. Conventional approaches to vaccine production are less effective, time-consuming, and expensive, so they are now almost obsolete. Designing vaccines through a relatively stable, safe, inexpensive, specific, and more practical immunoinformatics approach saves time and money [1, 21].

To develop a subunit vaccine against the *H. pylori* infection, this bacterium was used to identify target sequences using the protein advanced immunoinformatics approach. As mentioned earlier, the most detectable toxic agents in *H. pylori* infections are *APO*, *leoA*, *iceA1*, *iceA2*, *cag*, PaI, *vacA*, *cagþ*, *dupA*, *oipA*, *dupA*, and *babA*. The *lnt* (Apolipoprotein N-acyltransferase) gene encodes lipoproteins that line the cell [7–10]. Lipoproteins, a significant component of cell lining, contribute to several essential cell functions. In addition, they are generated by covalent linkage after lipid translations through a 3-stage sequential process directed by three integral membrane enzymes. These lipoproteins play vital roles, including nutrient uptake, protection of cell wall integrity, protein secretion, phytoplasma folding of proteins, and pathogenicity [7, 8]. The *leoA* (labile enterotoxin output A) gene is one of the pathogens of *H. pylori* GTPase, which is encoded in pathogenicity islands and potentially increases toxin release through secretory vesicles. *LeoA* is known as a bacterial diamine-like protein. Diamine family proteins alter membranes and generally evolve by releasing heat-sensitive enterotoxin through the membrane vesicle from the bacterial cells' surface. There are few reports on the functional role of bacterial diamine proteins [22, 23]. Membrane vesicles play an essential role in the protective function of the members of the diamine family.

Reports show this gene has the potential for immunogenicity in the host body. Therefore, it has been suggested as a suitable candidate for producing a gene vaccine against *H. pylori* [24]. The *H. pylori* *iceA* gene, induced in contact with the epithelium during bacterial attachment to the gastric mucosa, has two species (*iceA1* and *iceA2*). Evidence shows an association between *H. pylori* *iceA1* and peptic ulcers. There was a significant association between an iceA1 virulence factor and the *iceA1* gene with antibiotic mutations associated with clarithromycin. Most *iceA1*-positive isolates were mutant and resistant to clarithromycin. This issue confirms the relationship between resistance and the presence of virulence factor *iceA1* [21, 25, 26].

The immunogenicity of proteins was evaluated via the thorough analysis of online servers [27, 28]. Moreover, online tools were utilized to produce a rugged design for the subunit vaccine. Epitopes T-lymphocyte and B-lymphocyte were specified from the sequences of the selected proteins [29, 30]. T-cell receptors (TCRs) generally create an immune response to MHC-I and MHC-II molecules by activating T-lymphocytes via MHC-binding antigens or antigen-presenting cells (APCs). Based on the results of *the H. pylori* proteome immunoassay, the vaccine protein developed was able to cover a wide range of MHCs of classes I and II as well as linear B epitopes with similar physicochemical and structural attributes. Production of plasma and memory B cells would make it possible to protect the body against pathogen-related and other specific antigens [31, 32]. The final design of the vaccine included multiple binding epitopes of MHC I and MHC II that improved host immunity in the presence of adjuvants. Key parameters involved in the study were evaluated accurately to neutralize possible allergic reactions to the developed vaccine. Accordingly, the PSIPRED v3.3 server was employed for SS and 3D formations. The quality of the refined 3D model was verified using various servers. In addition, interactions between the designed vaccine and TLR-2, TLR-4, TLR-5, and TLR-9 were evaluated using the docking process, and docking complexes were stabilized using MD simulations. In this scientific study, immunoinformatic approaches confirmed the vaccine's effectiveness and stability.

Moreover, an agent-based model predicted an accurate response after vaccine sequencing. The vaccine designed in the present study was antigenic, non-allergenic, and effective in controlling *H. pylori* infections. Future clinical studies are recommended to assess the efficacy of this vaccine further. *H. pylori* is a cause of gastric infections and sometimes gastric cancer [33, 34]. This study aimed to design a novel multi-epitope subunit vaccine for the *H. pylori* antigen using a thorough immunoinformatics approach. To predict *the H. pylori* epitope, various immunoinformatics tools were employed. Several B- and T-cell epitopes were analyzed and combined with proper adjuvants and linkers to improve immunization of the new vaccine. According to the results, the vaccine had good antigenicity, solubility, allergenicity, tertiary structure, and physicochemical properties.

This study simulated molecular dynamics and binding of the vaccine and TLR-2, 4, 5, and 9. In addition, the binding stability and affinity of the TLK complexes and the vaccine were estimated. Besides, the cellular immune response to antigens was confirmed via immunoassay in silicon. The results obtained in this study were experimentally valuable, so they can be used in developing an experimental vaccine against *H. pylori*.

## Methods

### Collection of proteins

At this stage, we chose four proteins to produce an appropriate definition for the vaccine against *H. pylori*. The proteins included Apolipoprotein N-acyltransferase (*APO*), *leoA*, *IceA1*, and *IceA2*, all of which have been proven effective in managing the *H. pylori* infection. The most suitable proteins for designing a vaccine are those affecting the virulence of pathogens. Protein sequences were downloaded from the "protein" section of the https://www.ncbi.nlm.nih.gov/protein/ server with the GenBank IDs of ACX97396.1, ACX97803.1, AAL59391.1, and AAG49536.1.

### MHC-I binding epitope (CTL) prediction

The NetCTL 1.2 server, an online web tool (http://www.cbs.dtu.dk/services/NetCTL/), was used to predict CTL epitopes for four virulent proteins (*APO*, *leoA*, *IceA1*, and *IceA2*). The 9-mer epitopes were predicted using the introduced server for the query protein based on MHC-I binding, C-terminal cleavage, and transport-associated protein (TAP) scores. In this server, an artificial neural network is available for C-terminal cleavage, which bonds peptides to MHC-I, while the weight matrix could obtain tap transport efficiency. The foretoken threshold of epitopes was set at 0.75 [35].

### Prediction of MHC-II binding epitopes (HTL)

MHC-II binding was predicted for each of the selected virulent proteins (*APO*, *LeoA*, *IceA1*, and *IceA2*) by the web tool of IEDB MHC-II. These proteins were examined against a set of seven human leukocyte antigens (HLA), including HLA-DRB1 * 15:01, HLA-DRB4 * 01:01, HLA-DRB3 * 01:01, HLA-DRB5*01:01, HLA-DRB1 * 03:01, HLA-DRB3 * 02:02, and HLA-DRB1 * 07:01. The association between these peptides and MHC-II was defined based on the half-maximal inhibitory concentration (IC50). In addition, the value of IC50, less than 50, showed the highest dependency on MHC-II. Besides, the value of IC50 being less than 500 nM represented midrange dependence. Additionally, when the value of IC50 was less than 5,000 nM, it showed the lowest binding dependency. Score IC50 was inversely related to the percentile rank.

### B-cell epitope prediction

B lymphocytes (B cells) are crucial in empowering the immune system by secreting necessary antibodies for long-term immunity. Aimed to produce B-cell linear epitopes, the online server of BCPREDS (B-cell epitope prediction server) (http://ailab.ist.psu.edu/bcpred/) was used. This server predicted 20-mer linear B-cell epitopes using the kernel technique. The 20-mer linear B-cell epitopes of BCPred employed a backing vector machine (SVM) algorithm to predict B-cell (linear) epitopes [36].

### Vaccine sequence creation

A collection of HTL and CTL epitopes were chosen with the presupposition of having a major binding score and a non-allergenic nature. The selected CTL epitopes were linked to each other using an AAY linker to create an ultimate multi-epitope vaccine construct. On the other hand, the selected HTL epitopes were linked to each other using GPGPG linkers. The employed linkers improved epitope representation, and proper separation was achieved using them. There were two main reasons for using these linkers; firstly, they could successfully barricade the formation of functional epitopes (neo-epitopes) and could effectively improve epitope presentation [37–39]. Moreover, aiming at improving immunogenicity, the EAAAK linker was used as an adjuvant to add human β-defensins to the N-terminus of the vaccine. Linkers (AYY, KK, eaaak and GPGPG) play vital roles in producing an extended conformation (flexibility), protein folding, and separation of functional domains, and therefore, make the protein structure more stable [27].

### Allergenicity profiling

To ensure that the vaccine would not cause any allergic responses, the online antigenicity prediction server of AlgPred2.0 was used (https://webs.iiitd.edu.in/raghava/algpred2). The introduced server would use several algorithms (SVMc + MAST + IgEepitope + ARPs BLAST) to check the allergenicity of the inquiry sequence [40]. The results of this server are dependable, at the precision of 85% at the -0.4 threshold.

### Vaccine antigenicity profiling

One of the significant steps in designing a vaccine is to evaluate its antigenicity. In the present study, we used the two servers of Vaxijen 2.0 (http://www.ddg-pharmfac.net/vaxijen/VaxiJen/VaxiJen.html) and ANTIGENpro (http://scratch.proteomics.ics.uci.edu/) to predict antigenicity [41].

### Evaluation of physiochemical properties

Physicochemical properties of the vaccine sequence were evaluated using the online freely available web server of ProtParam (http://web.expasy.org/protparam/) [42]. The ProtParam server (References/Documentation) computed all required properties of GRAVY, the instability index, theoretical pI, in vivo half-life, in vitro half-life, the aliphatic score, molecular weight, and amino acid composition.

### Prediction of secondary structure

The secondary structure of the vaccine sequence was evaluated using the PSIPRED protein structure prediction server (http://bioinf.cs.ucl.ac.uk/psipred/) that provided results with high accuracy [27]. Besides, the PSI-Blast tool was used to identify proteins showing homology to our vaccine construct.

Next, these sequences were customized to create a position-specific scoring matrix (PSSM). Moreover, the introduced server utilized a neural network to process the PSSM and methodize secondary structure elements [43].

### D structure prediction of the vaccine

The I-TASSER (Iterative Threading Assembly Refinement) server is a homology modeling tool utilized for 3D or tertiary modeling of the multi-epitope vaccine (https://zhang.lab.ccmb.med.Umich.Edu/I-TASSER/). The I-TASSER server is an integrated platform used to predict and computerize the performance and structure of the protein. In addition, it utilized the sequence-to-structure-to-function paradigm to identify analogous structures from the Protein Data Bank (PDB)53n. Starting from an aminoalkanoic acid sequence, I-TASSER generated 3D atomic models from a wide range of repeated structural assembly simulations and threading alignments. Accordingly, a template modeling (TM) value less than 0.17 showed a random similarity, while a TM greater than 0.5 indicated a model with careful topology [44].

### D structure refinement of the vaccine

Validation of the 3D structure was refined using the three web tools of RAMPAGE47 (https://saves.mbi.ucla.edu/), ERRAT46, and ProSA-web45 (https://prosa.services.came. sbg.ac.at/prosa.php). The RAMPAGE server used PROCHECK principles for validation through investigating the Ramachandran plot. The ERRAT server worked based on the non-bonded interactions inside a given structure. Finally, Prosa-Web plotted general priority and underlined errors calculated in the 3D query. Next, UCSF Chimera software (www.rbvi.ucsf.edu/ chimera) was used to visualize the model [45–47]. Additionally, structure correction and energy minimization were performed using YASARA software.

### Evaluation of physiochemical properties

Physicochemical characteristics of the fusion protein were evaluated using a ProtParam software program (http://web.expasy.org/cgi-bin/protparam).

### The interaction analysis vaccine with TLR receptors

Human toll-like receptors of 2, 4, 5, and 9 (TLR-2, TLR-4, TLR-5, and TLR-9) and the vaccine were used. In addition, to conduct a thorough molecular docking study, the PatchDock server was used. Next, a candidate transformation test was done on complementary patched structures. Besides, their 3D structure was obtained from the RCSB PDB server (https://www.rcsb.org/) with IDs 6nig, 3fxi, and 3joa. Since toll-like 9 had no precise structure, its 3D structure was modeled and validated according to the abovementioned steps. Next, the transformations of every candidate were compared using the scores obtained through the geometric fit and its atomic desolvation energy. The input parameters for docking analysis were considered ligands and the PDB coordinate file for each protein. The root-mean-square deviation (RMSD) clustering was set at 4Å, and the complex type was modified to the protein–ligand type [48].

### Molecular dynamics simulation

According to the docking results, dynamic simulations were run to determine the effects and behavior of the vaccine for TLK-2, TLK-4, TLK-5, and TLK-9 receptors. Accordingly, MD simulations were performed by GROMACS 2020.1 software and force field G43a1. Besides, the SPC model was chosen for water molecules. Na + and Cl- ions were added to them to neutralize and eliminate undesired interactions. In addition, energy minimization was applied to the system for NVT particles in the system (N), the system's fixed volume (V), and temperature (T), in 300 0K, in 5 ns, and on the NPT system by the pressure of 1 bar, in 12 ns, and at 0.001 femtosecond time intervals. Besides, non-bond interactions were performed at 10 Å by the Particle Mesh Ewald (PME) method. Additionally, root mean square deviation (RMSD), root mean square fluctuation (RMSF), the radius of gyration, protein–protein hydrogen bonds, protein-solution hydrogen bonds, and the target protein’s secondary structure were obtained.

## Data Availability

All data are obtainable after an appeal from the corresponding author.

## Abbreviations

PME: Particle Mesh Ewald
RMSD: Root mean square deviation
RMSF: Root mean square fluctuation
PSSM: Position-specific scoring matrix
*H.** pylori*: *Helicobacter pylori*
MALT: Mucosa-associated lymphoid tissue
TCRs: T-cell receptors
APCs: Antigen-presenting cells
